# Dietary Approaches in Eosinophilic Esophagitis

**DOI:** 10.3390/nu18183053

**Published:** 2026-09-18

**Authors:** Tarek Arraf, Javier Chahuán, Luisa Bertin, Gianni Di Lecce, Emma Sirinic, Edoardo Vincenzo Savarino

**Affiliations:** 1 Gastroenterology Unit, Department of Surgery, Oncology and Gastroenterology, University of Padua, Via Giustiniani 2, 35128 Padua, Italy; arraf.tarek@gmail.com (T.A.); jnchahuan@gmail.com (J.C.); luluisa92@gmail.com (L.B.); giannidilecce97@gmail.com (G.D.L.); emma.sirinic@studenti.unipd.it (E.S.); 2 Faculty of Medicine, Technion Institute of Technology, Haifa 3525433, Israel; 3 Department of Gastroenterology, Rambam Health Care Campus, Haifa 3109601, Israel; 4 Departamento de Gastroenterología, Facultad de Medicina, Pontificia Universidad Católica de Chile, Santiago 8330077, Chile

**Keywords:** eosinophilic esophagitis, dietary therapy, elemental diet, food allergy, one-food elimination diet

## Abstract

Eosinophilic esophagitis (EoE) is a chronic, immune-mediated, non-IgE-mediated food allergy and a leading cause of dysphagia and food impaction in adults, with rising prevalence worldwide. Diagnosis requires ≥15 eosinophils per high-power field on esophageal biopsy after exclusion of other causes of gastrointestinal eosinophilia. Food allergens, principally milk, wheat, eggs, legumes and seafood, drive the inflammatory process, and treatment options include proton pump inhibitors, topical corticosteroids, biologic agents and endoscopic dilation for stricturing disease. Among these, dietary therapy is the only approach that removes the causative trigger rather than suppressing the inflammatory response, making drug-free, sustained remission achievable. Elemental diets achieve the highest histological remission rates but are poorly tolerated in practice; empiric elimination following a step-up strategy, starting with one or two foods and escalating in the absence of response, is more practical for most adults. Conventional allergy testing cannot reliably identify food triggers in EoE and is not recommended for this purpose. Reintroduction of the trigger food leads to relapse, making long-term adherence essential, and not all patients are suitable candidates: repeated endoscopy, dietary restriction, quality of life impact and the risk of avoidant restrictive food intake disorder (ARFID) are barriers that must be weighed before recommending this approach. This review summarizes the available dietary approaches in adult and adolescent EoE, covering empiric elimination strategies and their efficacy, allergy test-directed diets, patient selection, the role of diet alongside other treatments, and nutritional adequacy and adherence.

## 1. Introduction

Eosinophilic esophagitis (EoE) is a chronic, immune-mediated, food antigen-driven disease of the esophagus, characterized by symptoms of esophageal dysfunction and a histological finding of ≥15 eosinophils per high-power field (eos/hpf) on esophageal biopsy, in the absence of other causes of esophageal eosinophilia [1,2,3]. Once considered a rare entity, EoE has emerged over the past three decades as a leading cause of dysphagia and food impaction in adults, with global estimates placing the annual incidence at 5.31 per 100,000 inhabitant-years (95% CI: 3.98–6.63) and prevalence at 40.04 per 100,000 inhabitants (95% CI: 31.10–48.98) [4]. This rise exceeds what can be attributed to improved diagnostic awareness alone, implicating environmental and lifestyle factors in disease pathogenesis [5]. In adults, the clinical course is characterized by a progressive transition from an inflammatory to a fibrostenotic phenotype, with duration of untreated disease representing a predictor of esophageal stricture formation [6,7].

The central role of dietary antigens in EoE pathogenesis provides the mechanistic rationale for dietary therapy. The observation that an exclusive amino acid-based elemental formula—free of intact food proteins—induces histological remission in the vast majority of patients constitutes a proof of concept that food antigens, rather than aeroallergens or intrinsic esophageal factors, are the primary disease drivers [8,9]. Upon food reintroduction, histological relapse follows, supporting the causal role of dietary antigens. This food-antigen dependency positions dietary elimination as a treatment strategy that targets the root cause of disease rather than suppressing inflammation.

The current management of adult EoE relies on three main treatment approaches: pharmacological therapy, dietary therapy, and endoscopic dilation [1,10,11,12]. Pharmacological options include proton pump inhibitors (PPIs), swallowed topical corticosteroids, and the biologic dupilumab—a dual inhibitor of IL-4 and IL-13 signaling approved for EoE [13]. Endoscopic dilation targets strictures when present but does not modify the underlying inflammatory process [1]. Dietary therapy differs from pharmacological treatment in that it targets food antigens directly and can identify individual triggers to inform long-term management; however, it requires patient motivation and commitment and close dietetic support, and it is not suitable for all patients or all clinical cases.

This narrative review examines the evidence for dietary approaches in adult and adolescent EoE, from empiric elimination strategies to elemental formulas, and discusses how to integrate them into clinical practice. Alongside efficacy, it addresses the barriers that determine whether dietary therapy succeeds: adherence to prolonged elimination, the consequences of food restriction on eating behavior and quality of life, and the risk of food avoidance.

## 2. Pathophysiological Rationale for Dietary Therapy

The pathogenesis of EoE is multifactorial and involves an interaction between genetic predisposition and environmental exposures. Genome-wide association studies have identified susceptibility loci in genes encoding epithelial structural and immune-signaling proteins, suggesting that epithelial vulnerability precedes disease onset [14]. This genetic background alone, however, is insufficient to explain the sharp rise in EoE incidence observed over recent decades. Environmental factors—early-life antibiotic exposure, infant proton pump inhibitor or acid-suppressant exposure, caesarean delivery, reduced microbial diversity, and exposure to detergents and airborne allergens—have been shown to disrupt epithelial barrier integrity and promote immune sensitization [15,16,17,18]. When the esophageal epithelium is exposed to specific food proteins, antigen-presenting cells activate a Th2-polarized inflammatory cascade characterized by the release of IL-5, IL-13, and eotaxin-3. IL-5 drives eosinophil differentiation and survival, while eotaxin-3—overexpressed by esophageal epithelial cells—acts as the principal chemokine responsible for eosinophil recruitment to the esophagus [19]. IL-13 plays a particularly central role: it directly disrupts epithelial architecture, impairing barrier integrity and allowing greater antigen penetration—sustaining the immune response in a self-reinforcing cycle [16]. Mast cells contribute further by reducing epithelial resistance and amplifying barrier dysfunction, even in the absence of dense eosinophilic infiltration [20].

## 3. Empiric Elimination Diets

### 3.1. Elemental Diets

Elemental diets consist of formulas free of allergens, composed of free amino acids rather than intact proteins [21]. In 1995, Kelly and colleagues reported that in 10 symptomatic children with an eosinophilic infiltration of the esophagus attributed to gastroesophageal reflux (before the current diagnosis criteria of EoE), six of whom had already undergone fundoplication, following an elemental diet for at least 6 weeks led to resolution or improvement of symptoms and a reduction in peak intraepithelial eosinophil counts [22]. Subsequent studies confirmed the benefit of elemental diets in EoE [21,23,24,25].

A systematic review and meta-analysis of 465 patients found histological remission in 94.5% (95% CI: 92.3–96.4%), although the majority of patients were children, but without significant differences between children and adults [26,27]. Although most patients achieve remission with an elemental diet, not all do, suggesting that other antigens, such as aeroallergens, may trigger EoE [28].

Despite the high remission rate with elemental diets, they remain difficult to implement due to low palatability, nutritional risks, high costs and non-universal insurance coverage, poor adherence, monitoring by a dietitian, negative impacts on social activities, and related social restrictions [1,27]. Currently, guidelines do not consider this approach a standard dietary therapy, reserving it only for carefully selected patients with refractory disease [29]. A randomized trial in adults found that adding an amino acid formula to a four-food elimination diet (4-FED) did not improve histological remission compared with 4-FED alone but significantly improved disease-related quality of life [30].

### 3.2. Six-Food Elimination Diet (6-FED)

In 2006, Kagalwalla et al. published a retrospective study on empiric food elimination in children with EoE refractory to proton pump inhibitors (PPIs). Children were excluded from milk protein, soy, eggs, wheat, peanut/tree nuts, and seafood for 6 weeks. In total, 74% of patients in the 6-FED group achieved ≤10 eos/hpf in esophageal biopsies [31]. Following remission, individual foods were reintroduced sequentially with repeat endoscopy after each reintroduction. Cow’s milk was the most common trigger food, followed by wheat, and some patients had more than one trigger food. The entire reintroduction process took between 5 and 48 months, and the mean number of endoscopies was 5.6 [32].

Gonsalves et al. prospectively evaluated the 6-FED in adults with EoE. After a 6-week treatment period, 32 out of 50 patients (64%) had ≤5 eos/hpf and 37 out of 50 (74%) ≤15 eos/hpf in esophageal biopsies, along with improvement of dysphagia. In patients with reintroduction of trigger foods, symptoms recurred after a median of 3 days, with the most common triggers being wheat and milk [33].

In Spain, a study by Lucendo et al. using a 6-FED with the exclusion of cereals (wheat, rice and corn), milk, eggs, fish/seafood, legumes/peanuts, and soy in 67 adult patients with EoE found that after dietary exclusion 73.1% of patients achieved <15 eos/hpf. In total, 35.7% of patients had only a single trigger food, but one in three patients had three or more trigger foods identified. The most common trigger was cow’s milk. Patients who adhered to the exclusion of trigger foods maintained clinical and histological remission for up to 3 years [34].

A systematic review and meta-analysis including 22 studies with almost 1000 patients on 6-FED found that the effectiveness in achieving histological remission, defined as <15 eos/hpf, was 63.9% (95% CI: 58.5–69.2%; I^2^ = 63.6%), with comparable rates in children (67.5%) and adults (63.5%) [26]. Although approximately two-thirds of patients achieve remission with the 6-FED, this approach has several limitations that make adherence challenging, including the large number of endoscopies required to identify trigger food(s); the restrictive nature of the diet, with multiple foods excluded; and the prolonged duration of the process. In addition, most patients have fewer than three trigger foods [34], which prompted the development of simpler strategies [24,26,31,32,33]. Notably, pollen-sensitized adults with EoE showed a significantly lower response to the 6-FED during the pollen season compared with outside of it (21.4% vs. 77.3%; *p* = 0.003), suggesting that aeroallergen exposure may modulate dietary treatment outcomes [35].

### 3.3. Four-Food Elimination Diet (4-FED)

In a multicenter European study, the 4-FED was evaluated in adolescent (>14 years) and adult patients with EoE who had failed an 8-week trial of PPIs, eliminating dairy, wheat, eggs, and legumes. After 6 weeks of exclusion, 28 out of 52 patients (54%) achieved clinico-histological remission, and 31% of nonresponders achieved remission after escalation to a 6-FED [36]. In children, a 4-FED excluding milk, wheat, eggs, and soy achieved remission in 64%, with cow’s milk as the most common trigger [37]. A meta-analysis of seven studies including 329 patients with EoE treated with a 4-FED found a histological remission rate (<15 eos/hpf) of 54.7% (95% CI: 45.7–63.6%; I^2^ = 57.7%), with comparable rates in children and adults [26].

### 3.4. Step-Up Approach

Given the large number of endoscopies and the time required to identify trigger food(s), a step-up strategy starting with one or two food groups may reduce the number of endoscopies and shorten the diagnostic process compared with a top-down approach [38,39]. Since milk and wheat are the most common food triggers in EoE, a prospective study conducted in Spain and Italy evaluated the effectiveness of a step-up dietary approach in adult and pediatric patients with EoE. The protocol initially eliminated two food groups (milk and gluten-containing cereals) for six weeks, targeting histological remission (<15 eos/hpf). Nonresponders were offered a 4-FED, with the additional exclusion of eggs and legumes, followed by a 6-FED, which also excluded nuts and fish/seafood, if no response was achieved [38]. In patients who achieved remission, food groups were reintroduced individually, with an endoscopy performed after each reintroduction [38]. After the exclusion of milk and gluten-containing cereals, 43% of patients achieved remission [38]. Among nonresponders to the two-food elimination diet (2-FED), 54 out of 74 patients proceeded to a 4-FED, of whom 10 (18%) achieved clinico-histological remission. Among nonresponders to 4-FED, 27 out of 44 proceeded to a 6-FED, with eight (29%) achieving clinico-histological remission. Cumulative per-protocol remission rates after the two-, four- and six-food diets were 43%, 60% and 79%, falling to 43%, 54% and 68% when missing data were imputed for the whole enrolled cohort. Milk was the most common food trigger [38]. This approach reduced the number of endoscopic procedures by 20% and shortened the time required to complete the diagnostic process, potentially improving adherence [38].

### 3.5. One-Food Elimination Diet (1-FED)

Milk and dairy products are the most frequent food triggers in EoE [40]; milk elimination alone has therefore been evaluated as a first-line strategy. Adult patients with EoE who did not respond to PPIs were randomized to either a 1-FED excluding animal milk or a 6-FED excluding animal milk, wheat, eggs, soy, nuts, and seafood for six weeks. Patients who did not respond to the 1-FED were offered a 6-FED, whereas those who did not respond to the 6-FED were offered swallowed fluticasone [41]. After six weeks, 23 out of 67 patients (34%) in the 1-FED group and 25 out of 62 patients (40%) in the 6-FED group achieved histological remission (<15 eos/hpf), with no significant difference between groups. Among 1-FED non-responders who escalated to a 6-FED, nine out of 21 (43%) achieved histological remission [41]. A randomized trial of 63 children (aged 6–17 years) also compared 1-FED with 4-FED, finding similar histological remission rates (41% vs. 44%, respectively) [42]. A 2024 systematic review and meta-analysis of seven studies in 224 patients found histological remission in 46.4% (95% CI: 40.0–52.9%; I^2^ = 49.8%) [26]. More recently, a prospective multicenter study of 31 adults with EoE found that a 12-week dairy-free diet achieved histological remission in 15 out of 28 completers (53.6%; 48.4% by intention-to-treat), with significant improvements in symptoms, endoscopic severity, and quality of life [43].

### 3.6. Food Elimination, Reintroduction and Trigger Identification

Dietary therapy follows three phases: an elimination phase to induce remission, a reintroduction phase to identify the food or food groups that trigger the disease, and a maintenance phase in which the identified triggers are avoided [39,44,45,46]. Symptom improvement alone does not confirm histological remission, and histological evaluation should be performed after 6–8 weeks of the elimination therapy [39,45,46,47].

During the elimination phase, patients should strictly avoid the selected food or food groups and should be aware of the risk of cross-contamination or accidental ingestion, as the amount of food required to trigger the disease remains unknown [39,48]. In some patients, six weeks of dietary therapy may be insufficient and a longer period of restriction, such as 12 weeks, may be necessary. This may be particularly relevant when there is a trend toward improvement at six weeks or when contamination is present or suspected [39,49]. Common causes of nonresponse during the elimination phase include intentional or unintentional dietary nonadherence, cross-contamination, and incomplete or inadequate elimination of potential food triggers. Once these factors have been ruled out, patients may be offered a more restrictive dietary approach, taking their preferences into account, or switched to pharmacological therapy [39].

During the reintroduction phase, one food group should be reintroduced at a time while the remaining excluded foods are avoided [1,2,44]. Esophageal biopsies should be obtained after each reintroduction step to confirm the histological response; ACG guidance recommends an interval of 6–8 weeks per food before endoscopy [1]. Food reintroduction should begin only after remission has been confirmed during the elimination phase. Reintroduction should proceed from foods least to most commonly associated with EoE: EoeTALY recommends starting with seafood, followed by nuts, soy, eggs, wheat, and milk last [2], though the order may also reflect the patient’s preferences [39]. If histological remission is maintained after reintroducing a food, that food is not a trigger and may be safely included in the diet. If histological activity recurs, the food is confirmed as a trigger and should be re-eliminated; a washout period of 4–6 weeks is then recommended before reintroducing the next food group [2,39]. Once all triggers have been identified, the maintenance phase begins with continued avoidance of confirmed culprit foods, and adherence should be assessed periodically [50].

Across 11 studies including 387 patients treated with a 6-FED or 4-FED, the most common food triggers of EoE were dairy products (70.5%), followed by wheat (48.0%), eggs (27.6%), and legumes (20.7%) [40], though the composition of this category varies across primary studies—typically soy and/or peanut, with lentils and chickpeas included less [34,36]. In an analysis of 240 patients from eight studies evaluating a 6-FED, nuts and seafood were identified as triggers in 21.9% and 15.6% of patients, respectively [40]. These findings are relevant because many patients have more than one food trigger.

To reduce the endoscopic burden, less invasive alternatives have been explored. In a prospective study, 22 adults with EoE who had responded to a six-food elimination diet (fish, nuts, eggs, soy, wheat and milk) or, if non-responders, an extended eight-food elimination diet additionally excluding corn and legumes, underwent sequential food reintroduction guided by esophageal sponge cytology. This procedure required no sedation and was not associated with significant adverse events. However, agreement with esophageal biopsy was only 59% using a cutoff of <15 eos/hpf. In 32% of cases, sponge cytology was negative while the biopsy was positive, suggesting that active disease may go undetected with this approach [51].

Empiric elimination works best in patients who are prepared for the commitment that reintroduction and repeated endoscopy require. Starting with one or two foods and escalating only if remission is not achieved reduces the burden for most patients without meaningful loss of efficacy. Throughout all three phases, dietetic support is essential to ensure safe elimination, adequate nutritional intake, and the sustained adherence on which the intervention depends.

## 4. Allergy Test-Directed (Targeted) Elimination Diets

Dietary elimination of disease-triggering allergens offers the prospect of a long-term, drug-free and sustainable treatment. Because EoE is a food antigen-triggered disease, it was hypothesized that non-invasive food antigen testing might identify dietary triggers in advance, thereby avoiding broad empiric restrictions and reducing time, costs and patient burden [52]. Targeted, or allergy test-directed, diets use skin prick testing (SPT), atopy patch testing (APT) and food-specific serum IgE (sIgE), singly or in combination [52,53]. An early meta-analysis of dietary interventions appeared to support the strategy, reporting histological remission in approximately 45% of patients on a test-guided diet, with lower efficacy in adults than in children [54].

SPT and sIgE detect immediate, IgE-mediated hypersensitivity, whereas EoE behaves as a delayed, cell-mediated disorder [53]. Consistent with this, a randomized, double-blind, placebo-controlled trial in adults found that omalizumab reduced neither symptoms nor esophageal eosinophilia, while the same patients showed granular IgG4 deposits, abundant IgG4-bearing plasma cells and food-reactive serum IgG4, characterizing adult EoE as IgG4-associated rather than IgE-mediated [55]. APT, which assesses delayed-type hypersensitivity, appeared better matched to this pathophysiology, and the most favorable outcomes with APT have been reported in pediatric cohorts, in which an SPT/APT-guided diet achieved histological remission in 53% of children [56].

In adults the results have been consistently poorer. A prospective Spanish cohort of 22 patients following a diet directed by SPT, prick–prick testing and APT achieved histological remission in roughly one quarter of the population [57]. A retrospective North American cohort of 31 adults compared elimination guided by patient-reported allergens and SPT against empiric six-food elimination: remission rates, defined by fewer than 15 eos/hpf, were 32% and 56% respectively, a difference that did not reach significance (*p* = 0.41), while mean peak counts fell from 78 to 43 eos/hpf (*p* = 0.004) [58]. A prospective open-label pilot study of APT with intact food products in adults found a sensitivity of 5.88% (95% CI: 0.31–30.76) and a specificity of 92.0% (95% CI: 72.49–98.60); half the cohort had a positive patch test, yet only 16% had a result confirmed histologically on food reintroduction [59]. Applying five testing modalities in parallel to a single adult cohort, Philpott et al. found that none predicted food triggers and concluded that exclusion and rechallenge with histological reassessment remains the only dependable investigation [60].

The pooled efficacy of allergy testing-directed elimination is 39.5% (95% CI: 30.3–49.2) across pediatric and adult populations, the lowest of all dietary strategies, with marked heterogeneity (I^2^ = 78.8%); in adults it falls to 26.4%, although the difference from children (45.7%) did not reach statistical significance (*p* = 0.063), and summary estimates are indistinguishable across SPT, APT and sIgE [26,61]. Collectively, a mechanistic mismatch, poor predictive accuracy in adults and the absence of any efficacy advantage over empiric regimens have led to a decline in the use of allergy test-guided elimination in adult practice, with current guidelines and consensus documents endorsing empiric elimination strategies instead [1,2].

The poor results in adults are not surprising given that skin prick tests and specific IgE detect IgE-mediated sensitization, while EoE is driven by a delayed, cell-mediated response to food antigens. A positive allergy test does not confirm a trigger in EoE, and a negative one does not exclude it. For this reason, allergy test-directed elimination is not recommended as first-line therapy in adults, though it may have a role where empiric elimination is not feasible or where a concurrent IgE-mediated allergy is present.

## 5. Patient Selection for Dietary Therapy

Proton pump inhibitors, topical corticosteroids, empiric elimination diets, biologic therapy and endoscopic dilation are all endorsed as treatments for adult EoE [1,62]. The key practical question, therefore, is which patients are appropriate candidates for dietary therapy at all [63].

The principal advantage of dietary therapy, and a major reason for its appeal to patients, is its potential to identify the specific food or foods that initiate and sustain the disease. Once established, EoE food triggers appear to remain stable over time [64,65], allowing patients to exert a degree of control over disease remission and, in the event of relapse, to regain remission by improving adherence to the prescribed diet. As with many chronic conditions, however, adherence tends to wane as patients experience symptomatic improvement [66]. Several considerations should be weighed when selecting candidates: motivation; personal preferences and values; age and life stage; lifestyle; work and home circumstances; nutritional status; willingness to undergo repeated endoscopic assessment; and, where relevant, financial resources and coverage for multiple endoscopies and dietitian input [1,2,39].

Ideal candidates should be motivated patients who have access to a dietitian experienced in EoE and to structured written guidance on label reading and cross-contamination, who accept the endoscopies that supervised reintroduction requires, whose phenotype is predominantly inflammatory, and who have no comorbidity that makes food restriction hazardous [27,39]. Dietary therapy is generally poorly suited to patients in whom adherence is unlikely, nutritional reserves are marginal, restrictive eating patterns risk being reinforced, advanced fibrostenotic disease necessitates endoscopic dilation, or rapid symptom relief is a priority [27,39,67].

### 5.1. Step-Up Versus Step-Down

As detailed in Section 3, a step-up approach, beginning with one or two food groups and escalating in the absence of response, reduces endoscopic procedures and diagnostic time by 20% compared with starting at six foods [38], and a randomized controlled trial found equivalent histological remission rates between one-food and six-food elimination at six weeks [41]. These data support dietary therapy with the least restrictive regimen, thereby minimizing unnecessary dietary exclusions and reducing the number of required endoscopic procedures [2].

Step-down strategies, by contrast, start broadly and progressively liberalize the diet after remission, allowing systematic food reintroduction. When choosing between the two, clinicians should clarify which food groups are added or removed at each stage. In a step-up protocol, failure of an initial milk- and wheat-free diet typically leads to the addition of further restrictions rather than a switch of focus, so that the patient advances to a regimen eliminating milk, wheat, soy and eggs. This cumulative pattern of restriction can be more discouraging for some patients than a top-down approach, in which a broader elimination is gradually relaxed over time. At every step, clinical follow-up should include systematic assessment of nutritional adequacy and health-related quality of life, with adjustments to the dietary plan as needed; ultimately, the choice should be made through shared decision-making, incorporating patient preferences, tolerance for restriction and willingness to undergo repeated endoscopy [44,68].

### 5.2. Predictors of Dietary Response

Reliable predictors remain scarce, and there is currently no allergy test [26,60]. Among clinical variables examined in adults, a higher pre-treatment endoscopic reference score predicted histological non-response to six-food elimination, whereas symptom severity did not [69]. Aeroallergen exposure is a further modifier: in pollen-sensitized adults, six-food elimination was markedly less effective when undertaken during the pollen season than outside of the pollen season (3/14 [21.4%] vs. 17/22 [77.3%]; *p* = 0.003), and during the pollen season, pollen-sensitized patients also responded less well than non-sensitized patients (21.4% vs. 77.8%; *p* = 0.01) [35]. The most robust predictor, however, is behavioral rather than clinical. In a study by Haasnoot et al. on 177 adults, poor adherence to maintenance therapy (41.8% overall) was independently predicted not by the restrictiveness of the regimen but by low-necessity beliefs, that is, the conviction that treatment is not needed (OR: 4.42, 95% CI: 2.17–9.02; *p* < 0.001), which outweighed age under 40 years (OR: 2.57, 95% CI: 1.20–5.53), longer disease duration and greater symptom severity [66] (Table 1).

Beyond individual predictors, a range of patient-, treatment-, and disease-related barriers can limit the feasibility and sustainability of dietary therapy in real-world practice (Table 2; see also Section 7).

## 6. Dietary Therapy Alongside Other Treatments

The management of adult EoE comprises dietary therapy; pharmacological agents, principally proton pump inhibitors (PPIs), swallowed topical corticosteroids (STCs) and dupilumab; and endoscopic dilation for stricturing disease [1,2,75]. No randomized trial has compared diet directly with any pharmacological agent in adults, and the role of dietary therapy relative to other treatments is therefore defined by indirect evidence and shared decision-making [1,75,76].

PPIs induce histological response in approximately half of adults [10,76,77,78,79]. Evidence on their combination with dietary therapy is limited to a single randomized trial, conducted in children, which found a numerically higher remission rate when a 4-FED was added to omeprazole, though the difference was not statistically significant and withdrawal rates were higher in the combination arm [76,79]. In adults the evidence is retrospective: among 74 patients refractory to both monotherapies, 24 attempted the combination and 17 responded [80].

STCs are the usual comparator for dietary therapy. In a Cochrane review of 41 randomized trials including 3253 participants, the authors found that STCs produced a large histological benefit over placebo (RR: 11.94, 95% CI: 6.56 to 21.75; number needed to treat: 3; high certainty). The symptomatic benefit was smaller and less certain (RR: 1.74, 95% CI: 1.08 to 2.80; low certainty). Withdrawals for adverse events were in fact slightly fewer with corticosteroids (RR: 0.64, 95% CI: 0.43 to 0.96), so tolerability does not distinguish STCs from diet [76,81]. Direct evidence on the combination comes from a series of 23 adults and adolescents, 90% of whom had already failed diet or steroids alone. Adding STCs to dietary elimination produced symptomatic improvement in 88% at four months and reduced furrows from 84% to 55% (*p* = 0.02). Peak eosinophil counts did not fall significantly (54 to 36 per high power field; *p* = 0.12) and had returned to baseline by 30 months [82]. Combination therapy may therefore improve symptoms and endoscopic appearance without achieving histological remission, a distinction relevant when combination therapy is used as a bridge to de-escalation.

A network meta-analysis of 13 randomized trials in adolescents and adults did not include dietary elimination because no RCT connects it to the network, and the authors call for head-to-head trials to define sequencing [83].

Dupilumab was effective with and without concurrent diet [13] and irrespective of prior diet or baseline PPI use [84], although every comparison involving diet was graded as very low certainty [76]. Its combination with dietary therapy has also been examined in the opposite direction, as a means of enabling food reintroduction. In an open-label pilot of 21 patients aged 6 to 25 years who had all previously failed reintroduction, dupilumab permitted successful reintroduction in 86% of attempts, with no significant change in peak eosinophil count (*p* = 0.36) [85]. The population was predominantly pediatric, and replication in adults is required. Dupilumab lowers food-specific IgE, and loss of tolerance, manifesting as new IgE-mediated reactions on reintroduction, has been reported [86]. Other biologics under active investigation in phase 2 and 3 trials include cendakimab [87], benralizumab [88], lirentelimab [89] and etrasimod [90].

Dietary elimination remains the only approach that removes the trigger and permits drug-free remission, and it avoids the candidiasis and adrenal insufficiency documented with STCs [91,92,93]. It can be sequenced after PPI failure, combined with steroids, or maintained in patients who wish to limit pharmacotherapy.

## 7. Nutritional Adequacy, Quality of Life, and Adherence

Multi-food elimination removes several of the densest sources of protein, calcium, riboflavin and vitamin B12, and risk scales with the number of foods withdrawn. Dietary intake in this population departs from recommendations before any elimination is prescribed. In Dutch adults assessed at baseline, saturated fat intake exceeded and fiber intake fell below dietary reference values in both sexes. Reference values were not met for potassium, selenium, and vitamins A and D, and diet quality scores were lower than in the general population. The authors characterize this profile as pro-inflammatory [94]. These are reported intakes rather than biochemically confirmed deficiencies. Half of these patients already avoided fruit and vegetables because of pollen food syndrome, food allergy or EoE itself [94]. Dietary composition may also relate to disease activity: in 39 adults, intakes of protein, fat and micronutrients (zinc, vitamin B12 and folate) were inversely correlated with peak eosinophil count, though these associations are cross-sectional, uncorrected for multiplicity, and partly explained by reverse causation [95].

Involvement of a dietitian with expertise in food allergy is integral rather than adjunctive, and consensus guidance holds that with such support dietary therapy can be nutritionally balanced [39,74,96]. However, the 2–4–6 step-up trial enrolled 130 patients across 14 centers and remains the reference for step-up dietary therapy, yet it states that no registered dietitian or nutrition specialist was involved [38].

Quality of life is sparsely reported in the dietary trial literature [76,97]. In the SOFEED trial (Kliewer et al.), quality of life did not differ between 1-FED and 6-FED in 129 adults (mean difference: 0.57, 95% CI: −3.25 to 4.39); eliminating five additional foods conferred no measurable burden or benefit [41,76]. Serious adverse events were absent and withdrawals were few in both arms [76]. In the budesonide orodispersible tablet trial, the only quality of life domain to improve significantly over placebo was eating and diet impact (0.50, 95% CI: 0.17 to 0.82), while the overall score did not [98,99]; the domain most burdened by dietary therapy was thus the one most responsive to pharmacotherapy.

Restrictive eating is a recognized psychosocial risk of dietary therapy. Among 289 adults with organic digestive disease, including EoE, 53.7% screened positive for avoidant restrictive food intake disorder. Those on a physician-directed diet reported greater fear of gastrointestinal symptoms and less interest in food, although the instrument overestimates the diagnosis where avoidance is partly adaptive [70]. In the only adult EoE series, comprising 12 patients, weight loss occurred in 75% and psychiatric comorbidity in 83% [71]. Adaptive eating behaviors are more prevalent: among 95 adults, 46.3% had high esophageal anxiety, 47.4% hypervigilance and 55.8% used compensatory mealtime behaviors, more frequently in active than in quiescent disease (76.8% versus 25.6%) [72,73]. These behaviors constitute a form of self-imposed dietary restriction [72], and a proportion of patients are therefore already restricted at presentation, which argues for nutritional assessment before rather than after a diet is prescribed.

Associated distress is under-recognized. Among 147 Dutch adults, anxiety and depression scores matched population norms, yet 36% had significant distress. Of those, 57% denied any problem and 16% were receiving care, and the odds of anxiety were threefold higher in patients aged 18 to 35 years [100].

Prolonged avoidance carries a risk specific to dietary therapy. Patients who had avoided a food for years had become sensitized to it and reacted immediately on reintroduction, including with anaphylaxis. Guidelines advise involving an allergist whenever loss of tolerance is a concern, irrespective of the duration of avoidance [1]. Allergist consultation and collaboration are also advised throughout dietary elimination and long-term EoE care, including insight on aeroallergen exposure and its potential impact on dietary elimination timing [1], within an integrated multidisciplinary team strongly recommended for diagnosis, treatment and follow-up [1,44]. The published cases are pediatric, and the risk in adults is uncharacterized, but reintroduction is the procedure by which food triggers are identified, so the concern applies to the step that makes the diet informative.

Long-term adherence constrains dietary therapy. Of 42 adults reaching the maintenance phase of a 6-FED, 43% were no longer following it; former users rated it less effective, all reported that social situations impeded adherence, and 64% reported diet-related anxiety [101]. Adherence beyond several years is sustained by approximately half of patients [64,65], and more than 80% would still recommend dietary therapy. When measured directly among 177 adults, 41.8% were poorly adherent, with no significant difference between drug and diet (*p* = 0.320). The strongest independent predictor was low belief in the necessity of treatment (OR: 4.42, 95% CI: 2.17 to 9.02), ahead of age under 40 years [66]. Perceived necessity rather than regimen burden therefore appears to be the modifiable determinant of adherence.

## 8. Emerging Approaches and Future Directions

The principal unmet need in dietary management is identifying a patient’s specific food trigger without repeated endoscopies. Conventional allergy tests cannot achieve this, a limitation discussed in Section 4, and attention has shifted to food-specific IgG4. Since EoE in adults is IgG4-associated rather than IgE-mediated [55], food-specific IgG4 is a more plausible marker: it is overexpressed in esophageal tissue [102], and positive IgG4 staining predicted greater need for second-line treatment in 78 adults (RR: 2.5, 95% CI: 1.0 to 6.6) [103]. Milk-specific IgG4 also correlated with response to milk elimination in the SOFEED trial [41]. Prospectively, an IgG4-guided diet achieved histological remission in 45% of patients [104,105], comparable to empiric milk elimination alone [40]. Two limitations apply: combining IgG4 with blood T-cell responses improves accuracy only to 53–75%, with randomized evaluation of an 18-food panel still awaited (NCT05543512) [96]; and IgG4 to milk and wheat is elevated not only in the esophagus but in plasma, throat swabs, stomach and duodenum [106], confirming that it is not an organ-specific marker. The throat swab finding is nonetheless intriguing, as it raises the possibility of trigger identification without endoscopy.

The esophageal microbiome offers a different perspective on dietary therapy: rather than simply removing a trigger, diet may also modulate the bacterial environment of the esophagus. Evidence is limited and mixed; in 30 adults, alpha diversity changed significantly only in those on dietary elimination, an effect attributed to food restriction rather than disease improvement [107]. Short-chain fatty acids support esophageal barrier function in vitro [108], but elimination diets reduce gut microbial diversity [108], a trade-off that remains poorly understood.

A further refinement of milk elimination has been proposed at the molecular level. Standard cow’s milk contains a mixture of A1 and A2 beta-casein isoforms, differing by a single point mutation (proline to histidine at position 67) that arose in modern cattle. In a preliminary prospective study of 11 adults with confirmed milk-induced EoE in histological remission, 63% maintained remission during a 12-week challenge with A2-only milk (300 mL/day), and ex vivo stimulation of esophageal biopsies with standard but not A2 milk elicited significantly greater IL-5 secretion, implicating A1 beta-casein specifically as the local inflammatory stimulus [109]. Endoscopic activity scores increased modestly in some patients despite stable eosinophil counts; the design was unblinded and no standard milk re-challenge was performed; replication in larger controlled trials is required before A2 milk substitution can be recommended as an alternative to complete milk elimination.

Direct mucosal provocation has been proposed as an alternative to prediction. Injection of six food allergens into the esophageal mucosae of eight adults produced an acute response in five and a delayed weal or flare in two more, with none in three controls [110]. A molecular readout of this response has since been described, with TNFSF18, also called GITRL, rising 12-fold above baseline 20 min after intramucosal injection [111]. With only thirteen patients in total, these findings are hypothesis-generating, but the approach is appealing: it assesses the esophageal mucosa directly rather than the skin and measures a provoked response rather than an associated marker.

Standardization remains a limiting factor. The AGREE consensus removed the PPI trial requirement, which altered the populations recruited into dietary studies [112]. The previously mentioned Cochrane review found heterogeneity in outcome definitions to be pervasive and called for validated instruments aligned with the COREOS core outcome set and, explicitly, for randomized placebo-controlled trials of dietary elimination [76]; no sham-diet-controlled study exists. Future adult trials should prespecify explicit handling of dietary and PPI co-interventions and should treat adherence and restrictive eating as core outcomes. The effect of context should also be accounted for. A 6-FED was substantially less effective during pollen season in adults sensitized to pollens (21.4% versus 77.3%) [35]. However, season had no effect in the unselected population of the 2–4–6 study (43% versus 43%, *p* = 0.8) [38].

## 9. Conclusions

Dietary therapy is the only EoE treatment that removes the causative trigger rather than suppressing inflammation, achieving drug-free, sustained remission [55]. Elemental diets achieve the highest remission rates but are poorly tolerated; a step-up empiric elimination strategy is more practical for most adults, with remission ranging from 34% with one-food elimination to 68–79% after escalation to six foods [38,41]. Milk, wheat, eggs and legumes are the most common triggers on systematic reintroduction [40]. Allergy testing predicts food triggers poorly in EoE and is not recommended for this purpose [1,26]; adherence over the long term, driven more by the patient’s belief in the need for treatment than by the number of foods excluded, is the main determinant of success [66,101].

The key remaining challenge is identifying individual triggers without repeated endoscopy. Food-specific IgG4, mucosal provocation and microbiome profiling are under investigation [55,108,110,111], and molecular refinement of milk elimination with A2 beta-casein remains an early hypothesis [109]; validated non-invasive tools could make dietary therapy more accessible and sustainable.

## Figures and Tables

**Table 1:** Patient selection for dietary therapy in adult EoE.

Good Candidate for Dietary Therapy	Poor Candidate for Dietary Therapy
High motivation and readiness to commit	Poor expected adherence
Access to an EoE-experienced dietitian	No access to specialist dietitian
Willingness to undergo repeated endoscopies	Unable or unwilling to undergo serial endoscopies
Predominantly inflammatory phenotype	Advanced fibrostenotic disease requiring dilation
Preference for drug-free, trigger-targeted treatment	Need for rapid symptom relief
No nutritional risk factors or comorbidities making restriction hazardous	Marginal nutritional reserves
Absence of pre-existing restrictive eating patterns	Pre-existing restrictive eating or ARFID

ARFID, avoidant/restrictive food intake disorder.

**Table 2:** Practical barriers to dietary therapy in adult EoE.

Domain	Barrier	Key Evidence	Clinical Implication
**Patient-related**	Pre-existing restrictive eating and ARFID	53.7% of adults with organic GI disease screened positive for ARFID; in the only adult EoE series (*n* = 12), weight loss in 75% and psychiatric comorbidity in 83%	A proportion of patients are already restricted at presentation [70,71,72,73]
	Meal-related anxiety and hypervigilance	Among 95 adults, 46.3% had food-related anxiety toward swallowing and 55.8% used compensatory mealtime behaviors, more frequent in active than quiescent disease	These behaviors constitute a form of self-imposed dietary restriction [72,73]
	Long-term adherence	43% no longer following 6-FED at maintenance; among 177 adults, low belief in treatment necessity was the strongest predictor of poor adherence (OR: 4.42), ahead of age, disease duration and symptom severity	Non-adherence driven by low-treatment-necessity beliefs, not by dietary burden [66]
	Sensitization and anaphylaxis on reintroduction	Cases of IgE-mediated sensitization and anaphylaxis reported after prolonged food avoidance	Guidelines recommend involving an allergist whenever loss of tolerance is a concern, regardless of avoidance duration [1]
**Treatment-related**	Repeated endoscopy requirement	Each reintroduction step requires endoscopic confirmation; step-up strategy reduces procedures by ~20% vs. starting at six foods [38]	Non-invasive alternatives to endoscopic assessment are under investigation but remain insufficiently validated for clinical use [1,51]
	Limited dietitian expertise	The 2–4–6 step-up trial (130 patients, 14 centers) reported no involvement of a registered dietitian	Dietitian involvement is considered integral, not adjunctive, by consensus guidance [1,2,39,74]
	No reliable food trigger predictor	No allergy test (SPT, APT, specific IgE) predicts food triggers reliably in adults	Exclusion and rechallenge with histological reassessment remains the only dependable investigation [60]
**Disease-related**	Fibrostenotic phenotype	Advanced stricturing disease requires endoscopic dilation; dietary therapy does not reverse subepithelial fibrosis	Dietary therapy is poorly suited to patients in whom fibrostenotic disease necessitates dilation [1,6]
	Pollen sensitization	6-FED efficacy fell from 77.3% to 21.4% during pollen season in sensitized adults (*p* = 0.003) [35]	In pollen-sensitized patients, the timing of dietary intervention relative to pollen season should be taken into account when planning and interpreting 6-FED [35]

ARFID, avoidant/restrictive food intake disorder; APT, atopy patch test; EoE, eosinophilic esophagitis; FED, food elimination diet; GI, gastrointestinal; IgE, immunoglobulin E; OR, odds ratio; SPT, skin prick test.

## Data Availability

No new data were created or analyzed in this study. Data sharing is not applicable to this article.
